# Sleep pathology and use of anabolic androgen steroids among male weightlifters in Norway

**DOI:** 10.1186/s12888-024-05516-6

**Published:** 2024-01-22

**Authors:** Sandra Klonteig, Morgan Scarth, Astrid Bjørnebekk

**Affiliations:** 1 SINTEF Digital, Box 124 Blindern, N-0314 Oslo, Norway; 2 Anabolic Androgenic Steroid Research Group, Section for Clinical Addiction Research, Division of Mental Health and Addiction, Oslo University Hospital, Oslo, Norway; 3 Department of Psychology, University of Oslo, Oslo, Norway

**Keywords:** Anabolic androgenic steroids, Sleep, Hormones, Testosterone, Androgens, Anxiety, Depression, Weightlifting

## Abstract

Use of anabolic androgenic steroids (AAS) causes drastic changes in hormonal milieu and is associated with a range of medical and psychological consequences. Sleep pathology is a common side-effect of AAS use but few have studied these relations. This study examined the relationship between AAS use, psychological distress and sleep quality, and how phases of heavy use and abstinence influence sleep. The Pittsburgh-Sleep-Quality-Index (PSQI) and Jenkins Sleep Scale (JSS) were used to assess sleep quality, and psychological distress was measured with the Hopkins Symptoms Checklist (HSCL). Participants comprised men who have previous or current long-term use of AAS (*n* = 68) and non-using weightlifting controls (WLC) (*n* = 58), where a subgroup of participants (*n* = 22) was monitored over ~ 6 months during phases of AAS use and withdrawal. Group differences on PSQI and JSS were evaluated with Kruskal-Wallis H tests, and the mediating role of psychological distress was evaluated using structural equation modeling. Linear mixed models were used to assess the role of AAS use and withdrawal on sleep quality. Among the AAS group, 66% reported sleep problems as a side effect, and 38% had used sleep medication. PSQI scores showed significantly lower sleep quality in the AAS group compared to WLC (*p* < 0.001) on all subscales except “sleep latency”. Furthermore, sleep quality was significantly poorer during withdrawal-phases than periods with AAS use (*p* < .001). Our findings provide key insight into sleep disturbances among men who use AAS, suggesting a link between sleep disturbances and hormone levels that deviate from physiologically normal levels in both directions.

**Supplementary Information:**

The online version contains supplementary material available at 10.1186/s12888-024-05516-6.

## Introduction

It is well established that steroid hormones influence sleep [1]. Strong correlations between natural hormonal fluctuations and sleep across species have repeatedly been shown [2], and thus behavior or substances that affect hormone levels may have consequences for sleep. Anabolic-androgenic steroids (AAS) are a family of hormones including testosterone and synthetic derivatives of testosterone and are commonly used by men to increase muscle mass for athletic or cosmetic purposes. About 2–3% of the general population in Europe and the USA report lifetime AAS use [3]. The prevalence is much higher, however, in certain subpopulations such as athletes (13.4%), recreational sportspeople (18.4%), and substance use patients (28%) [4, 5]. Sleep pathology is often mentioned as one of the reported side-effects of AAS use and is a common clinical observation [6–9]. About half of men who use AAS reported insomnia as a subjective side effect [8], and polysomnographic measurements show that men who use AAS have reduced sleep efficiency compared to controls [10]. Despite this, a comprehensive investigation of sleep quality in larger populations of men who use AAS is lacking.

Testosterone levels fluctuate in a diurnal rhythm; with high levels on awakening, declining during the day, and then rising again during sleep [11, 12]. A common practice of administering AAS is to alternate between heavy use and abstinence periods, lasting for several weeks or months. Doses taken are typically in a range that exceeds male production of testosterone by 5–100 times [13]. Such intake leads to high systemic androgen levels, and a subsequent suppression of the hypothalamic-pituitary-gonadal (HPG) axis due to negative feedback mechanism. A prolonged suppression of HPG may thus diminish endogenous testosterone production and cause hypogonadism upon abstinence, commonly referred to as anabolic steroid-induced hypogonadism (ASIH) [14]. Given that studies have linked hypogonadism to poor sleep quality [15], men who use AAS may be vulnerable to sleep disturbances upon abstinence (i.e. in the “off cycle”). Additionally, short-term administration of AAS has also been related to poor sleep quality [16]. However, the directionality and clinical significance of the associations between hypogonadism and poor sleep quality remains unclear [15]. Consequently, the impact of AAS use on sleep, including how different AAS administration patterns affect sleep, is still uncertain.

Sleep is a complex physiological process that plays an important role in numerous recovery processes and brain functions, and is closely linked with mental health. The relationship between sleep and mental health is bidirectional, with sleep pathologies being highly correlated with psychological disorders such as depression and anxiety [17]. For instance, primary depression disorders are associated with subsequent insomnia [18], while insomnia is a risk factor for the development of depression [19]. A recent meta-analysis highlighted the importance of sleep quality for mental health, as interventions that improve sleep quality also show medium effects on mental health outcomes. Long-term AAS use has been linked with psychiatric symptoms such as depression, anxiety and aggressive behavior [20, 21]. Therefore, it is relevant to investigate the impact of poor sleep and potential mediating effects of mental health problems in relation to AAS administration for the development of effective strategies for treating dependence and misuse of AAS.

In the current study we investigated sleep quality in relation to prolonged high-dose AAS use in male weightlifters. Specifically, we compared subjective sleep quality between men who have previous or current long-term use of AAS and a weightlifting control group, hypothesizing that sleep quality would be poorer among the AAS group as compared to the control group. In addition, we monitored sleep quality in a subsample of men who currently use AAS throughout different phases of AAS-administration, including both periods of AAS use and drug-free periods, to investigate the potential influence of hormonal status on sleep. Finally, we tested for associations between self-reported sleep quality and psychological distress.

## Methods

### Participants and data collection

The total study sample consisted of 126 adult male weightlifters, including 68 participants with current (*n* = 44) or past (*n* = 24) AAS use and 58 weightlifting controls (WLC). The study is part of a longitudinal study of the brain, medical and mental health consequences of long-term AAS use, carried out at the Anabolic Androgenic Steroid Research Group at Oslo University Hospital, Norway (https://www.ous-research.no/anabolic-steroids/). Participants were recruited through social media, online forums targeting people interested in heavy weight training, bodybuilding, and forums addressing AAS use. In addition, posters and flyers were distributed in select gyms in Oslo, Norway and surrounding areas. Inclusion criteria for the AAS group were previous or current AAS use corresponding to at least 1 year of cumulative AAS use. Inclusion criteria for the weightlifting controls (WLC) were being adult male engaged in heavy resistance training with no history of AAS use or other prohibited doping substances. The no-use-self-report was also objectively verified with antidoping drug screening, as reported in a largely overlapping sample [22]. We strived to match WLC to men who use AAS’ commitment to heavy strength training and targeted men who had managed to bench press 120 kg (∼265 pounds) for at least one repetition, where 100 kg (220 pounds) was the minimum criteria for inclusion. Exclusion criteria included self-reported history of severe head injury with loss of consciousness for > 1 min, a vascular or neurological disorder affecting the brain (e.g., history of diagnosed stroke, brain tumor, Parkinson’s disease, or epilepsy) and IQ < 80.

### The cycling sub-sample

To capture the influence of hormonal fluctuations during AAS exposure and withdrawal on sleep, a subsample of participants (*N* = 22) was monitored over a period of approx. 22 weeks. The sample comprise 11 men engaging in cyclic AAS administration, covering both phases of AAS-administration (“on-cycle”) and withdrawal phases (“off-cycle”), and 11 WLC. Similar criteria for inclusion and exclusion applied to this sub-sample, although slightly stricter as we aimed to isolate the effect of being on or off AAS. Additional inclusion criteria comprises; weekly AAS doses exceeding 300 mg/week in “on-cycle” periods, and where participants completely abstain from AAS for at least 4 weeks, including the use of low-dose testosterone delivered through transdermal routes (e.g. patches or gel). Additional exclusion criteria included severe mental illness as major depression, bipolar disorder or psychosis, use of prescribed medications for with known CNS effect and problematic use of alcohol and illegal drugs as defined by scores ≥5 on alcohol and drug use disorders identification tests (AUDIT-C, and DUDIT-C). As it was challenging to identify participants who met all the criteria, some of the criteria were relaxed during recruitment. Two participants in the WLC group and four in the AAS group presented AUDIT scores suggesting an increased risk of harmful alcohol use (scores above 8), however, none reached the threshold for high-risk alcohol consumption (scores above 16).

### Procedures and materials

The participants completed a set of structured questionnaires using a web solution offered by the Services for Sensitive Data provided by the University of Oslo. The questionnaires assessed relevant background and health information. The AAS group were also asked about their AAS use, and experienced side-effects of use. Intelligence Quotient (IQ) was assessed during neuropsychological testing, using the Wechsler Abbreviated Scale of Intelligence (WASI) battery [23].

Subjective sleep quality was examined with the Norwegian translated version of Pittsburg Sleep Quality Index (PSQI) questionnaire [24]. PSQI is a self-administered questionnaire containing 19 items which generates 7 components, namely subjective sleep quality, sleep latency, sleep duration, habitual sleep efficiency, sleep disturbances, use of sleeping medications, and daytime dysfunction over the last month. Each of the seven component scores is weighted equally on a scale from 0 to 3, and is subsequently summed to generate one global score. Global scores yielding more than 5 are considered to indicate poor sleep. One component (5j) allows for a description of “other reason(s)” for troubles following sleep. Unfortunately, due to technical issues we lack the weighted frequency response on the scale 5j from all participants’, but the written responses will be shortly summarized in the results section.

Jenkins Sleep Scale (JSS) was used to assess subjective sleep quality during the past month. The scale was originally designed to track common sleep problems in clinical populations. The questionnaire consists of four items, namely difficulty to fall asleep, wake up at night, difficulty to stay asleep and wake up exhausted in the morning instead of sleeping as usual. All items are presented with a six-point Likert scale: 0 (never), 1 (23 days), 2 (four – 7 days), 3 (eight to 14 days), 4 (15–21 days) and 5 (22–31 days). For the cycling study, this scale was adapted such that sleep quality was only assessed during the last 2 weeks. For that scale, the items were presented on a five-point Likert scale: 0(never), 1(1–2 days), 2(3–4 days), 3 (5–6 days) and 4(7 days). For both versions of the questionnaire, the sum of all the scores yields the total JSS score, which renders a potential score-range from 0 to 20. A total JSS score above 12 indicates clinical relevance with frequent sleep problems [25].

Depression and anxiety symptoms were measured using the Hopkins Symptom Checklist-25 (HSCL-25) and a shorter version, HSCL-10, for measurements during cycling [26]. The HSCL-25 consists of a 10-item subscale for anxiety and a 15-item subscale for depression, where each item is scored on a Likert scale from 1 (not at all) to 4 (extremely). A mean total score of 1.75 or above is a widely used cut-off for significant psychological distress.

### Statistical analysis

All statistical analyses were conducted using R version 4.0.3 (R Core team, 2019). A two-sided significance α-level was set to .05 in all analyses. Comparisons of the AAS and the WLC group in background information and global and subscale scores on PSQI and JSS were assessed with Kruskal-Wallis rank sum tests. Non-parametric tests were chosen due to the variables violating the assumption of normality, assessed with Shapiro- Wilk test (*p* < .05). No extreme outliers were detected. No further adjustment in sub- and sensitivity analyses were made, as the sample size is quite large and no large deviations were spotted upon visual inspection of QQ plots.

Correlations among the PSQI and HSCL scales were computed with Spearman’s rank coefficients (ρ). To investigate the potential influence of duration of AAS use, age at initiation, dose, and training, Spearman’s rank correlation coefficients were computed for each of these factors with HSCL, PSQI, and JSS sum scores. We fit a multiple mediation model using the Lavaan package in R, to assess indirect effects of AAS use on anxiety and depression on sleep, and direct effects of AAS use on sleep. We allowed anxiety and depression to correlate and adjusted for age on sleep. The statistical significance of the estimates and effects were derived from 95% confidence intervals based on 10,000 bootstrap samples to account for non-parametric values. Standardized estimates were used in the path model. WLC was used as a reference in the group variable. Sensitivity analyses were run to exclude a question in the HSCL-25 directly assessing “difficulty to sleep” see Fig. S2.

To assess how changes in state and hormonal fluctuations are associated with changes in the total JSS score, linear mixed effects (LME) models were applied using the lme function in the R (72) package lme4 (73). In fitting the model, we entered state (AAS on, AAS off and WLC) as a fixed effect. Participant ID was entered as a random effect (intercept). Visual inspection of residual plots did not reveal obvious deviations from homoscedasticity or normality. In a subsequent analysis, HSCL sum score was entered in the model as a fixed effect to assess the role of psychological distress. Sensitivity analyses were run to exclude a question in the HSCL-10 directly assessing “difficulty to sleep”, see Table S3 in appendix.

## Results

### Sample characteristics and characteristics of anabolic androgenic steroid use

Sample characteristics and characteristics of AAS use are presented in Table 1 and Table S1 (appendix) for the “cycling” subsample being monitored throughout different phases of AAS-administration. The groups did not differ in age, however years of education and IQ was higher among the WLC group, and the AAS group were heavier and stronger, as measured by bench press maximum. There was significant variation in years of AAS use (mean = 11 years, SD = 8) and debut age (mean = 23 years, SD = 8). The AAS group reported more lifetime use of prescribed- anxiety, sleep and antidepressant medication compared to WLC group in general. Two-thirds of AAS group reported sleeping problems of varying degrees as a side-effect of use. In addition, the AAS group demonstrated higher levels of depression and anxiety on the HSCL.

**Table 1:** Sample characteristics and characteristics of anabolic androgenic steroids (AAS) use

Variable	WLC, *N* = 58^1^	AAS, *N* = 68^1^	Test Statistic	*p*-value^2^	η^2^
**Age**	37 (9)	38 (11)	0.01	> 0.9	−0.008
**Edu**	16.76 (2.64)	14.95 (2.54)	15	< 0.001	0.114
**IQ**	117 (9)	108 (10)	21	< 0.001	0.158
**Height**	182 (6)	182 (7)	0.02	0.9	−0.008
**Weight**	93 (11)	100 (17)	5.8	0.016	0.039
**Strength training (min/week)**	382 (210)	356 (210)	0.40	0.5	−0.005
**Endurance training (min/week)**	101 (130)	100 (155)	1.0	0.3	0.000
**Bench max**	142 (22)	175 (35)	29	< 0.001	0.225
**Total years AAS use**	NA (NA)	11 (8)			
**Debut age**	NA (NA)	23 (8)			
**Current/previous AAS use**					
Current	0 / 0 (NA%)	44 / 68 (65%)			
Previous	0 / 0 (NA%)	24 / 68 (35%)			
**Anxiety medication**^3^	4 / 58 (6.9%)	15 / 66^4^ (23%)		0.023	
**Antidepressants**^3^	4 / 58 (6.9%)	17 / 66^4^ (26%)		0.007	
**Sleeping medication**^3^	4 / 58 (6.9%)	25 / 66^4^ (38%)		< 0.001	
**Sleeping problems as side-effect of AAS use**					
No problems	0 / 0 (NA%)	22 / 65^5^ (34%)			
Some degree	0 / 0 (NA%)	22 / 65^5^ (34%)			
Certain degree	0 / 0 (NA%)	17 / 65^5^ (26%)			
Large degree	0 / 0 (NA%)	4 / 65^5^ (6%)			
**HSCL total**	1.22 (0.28)	1.42 (0.43)	11.25	< 0.001	0.083
**HSCL anxiety**	1.15 (0.26)	1.36 (0.45)	10.61	0.001	0.078
**HSCL depression**	1.26 (0.33)	1.45 (0.45)	8.63	0.003	0.06

*AAS* anabolic androgenic steroids: *WLC* weightlift controls

^1^Mean (SD); n / N (%)

^2^Kruskal-Wallis rank sum test; Fisher’s exact test

^3^Prescribed medication

^4^missing data (*n* = 2)

^5^missing data (*n* = 3)

### Pittsburgh-sleep-quality-index and Jenkins sleep scale group comparisons and relations between scales

**Fig. 1:**
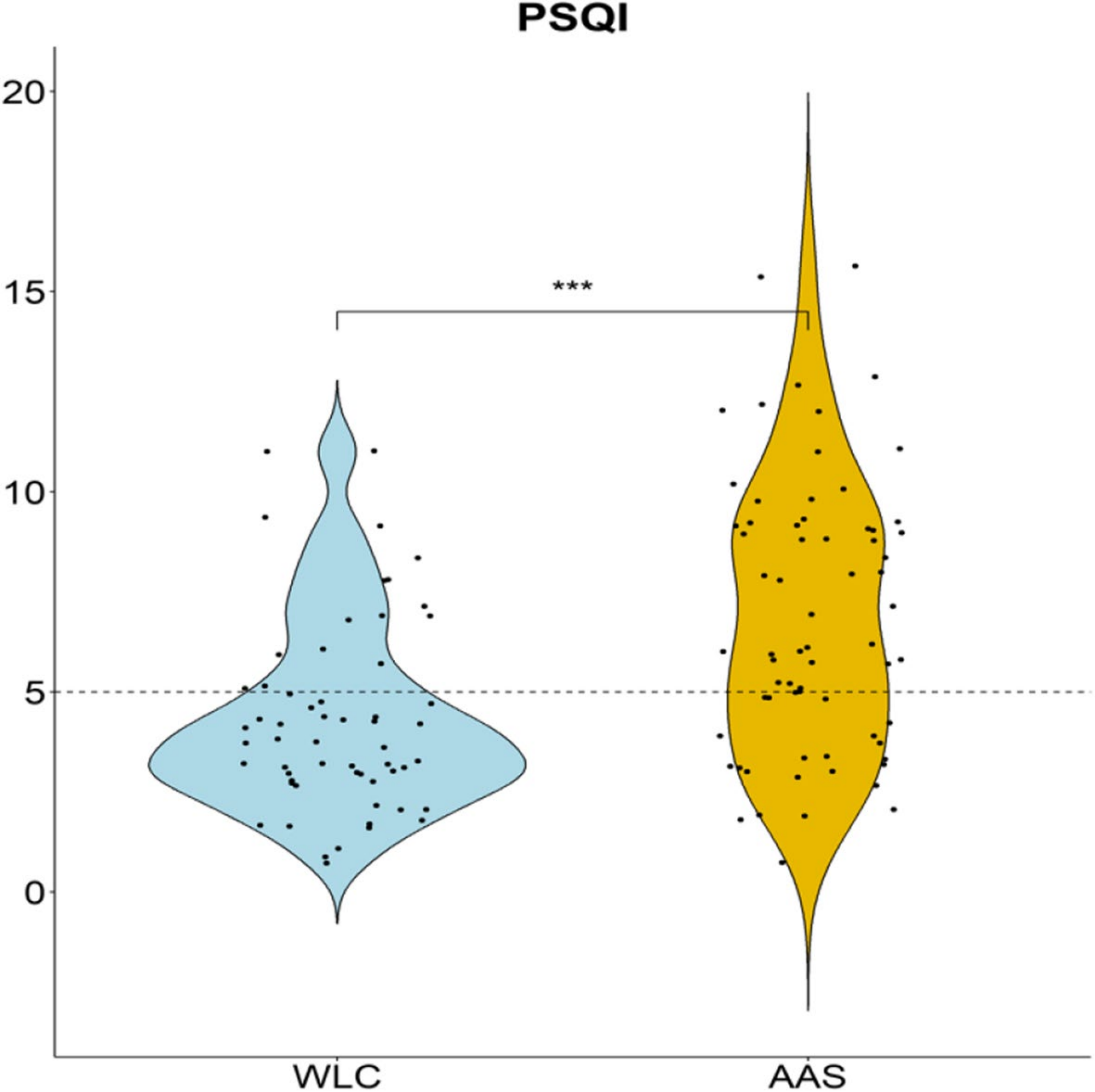
Distribution of PSQI scores among the AAS group and WLC. Y axis represents global PSQI score. Dotted line indicate cut-off value > 5. PSQI: Pittsburg Sleep Quality Index, WLC: weightlifting controls, AAS: anabolic androgenic steroid group

Global PSQI scores and subscales are presented in Table 2 and Fig. 1. Kruskal-Wallis rank sum tests showed significantly higher scores in the global PSQI for the AAS group as compared to WLC, and with a high proportion of the AAS group above the clinical cut-off. All subscales of the PSQI but the “sleep latency” component were significantly higher in the AAS group compared to WLC. Effect sizes for the different components ranged from small to large, with particularly strong relations found for the global PSQI and the “sleep medication” component. In addition, participants’ free-text responses regarding other types of sleep-related problems (PSQI item 5j) provided additional information related to psychological, lifestyle, physical and other factors that may impact sleep quality. Both AAS and WLC group reported similar findings, and the summary of results does not distinguish between groups. Psychological factors such as general stress, daily stressors related to personal life or work, nightmares, worries, relational problems, psychological pressure from relations, racing thoughts difficulty “turning off” the mind was reported. Lifestyle factors including work, particularly shift work, and caring for children were also frequently mentioned. Physical factors such as back pain, muscle cramps, general pain, low body fat percentage (especially before bodybuilding competitions), sleep apnea and other related issues were also reported. Additional reasons for poor sleep quality included substances that affect sleep, such as Ritalin and coffee, as well as direct links to AAS use as reported by some participants.

**Table 2:** Group comparisons of global Pittsburgh-Sleep-Quality-Index (PSQI) scores and subscales

Variable	WLC, *N* = 58^1^	AAS, *N* = 68^1^	Test Statistic	*p*-value^2^	η^2^
**Global PSQI**	4.34 (2.36)	6.85 (3.42)	19	< 0.001	0.141
**Sleep quality**	0.90 (0.67)	1.22 (0.67)	7.7	0.005	0.054
**Sleep latency**	0.98 (0.81)	1.24 (1.04)	1.6	0.2	0.005
**Sleep duration**	0.47 (0.60)	0.87 (0.96)	4.9	0.026	0.032
**Sleep efficiency**	0.31 (0.60)	0.71 (1.01)	5.1	0.024	0.033
**Sleep disturbances**	1.00 (0.42)	1.22 (0.51)	6.4	0.012	0.043
**Sleep medication**	0.10 (0.48)	0.69 (1.05)	17	< 0.001	0.133
**Daytime dysfunction**	0.59 (0.59)	0.91 (0.75)	6.1	0.014	0.041
**Global PSQI > 5**	20 (34%)	49 (72%)		< 0.001	

*PSQI* Pittsburgh-Sleep-Quality-Index

^1^Mean (SD); n (%)

^2^Kruskal-Wallis rank sum test; Fisher’s exact test

Significant group differences were also seen on sleep quality as assessed with the JSS, where the AAS group had higher JSS total score compared to WLC, with significantly higher scores on all JSS items. See Table S2 in appendix for comparisons of the total and subscales of the JSS between the AAS and WLC group.

Spearman correlation showed a strong relationship between the JSS total score and the global PSQI scores (rho = 0.72, *p* < .001). Contrary to the PSQI scale, only 16% of the AAS group were above the cut-off score indicative of serious disturbed sleep.

### Associations between anabolic androgenic steroid use, Pittsburgh-sleep-quality-index and Hopkins symptoms checklist anxiety and depression scores

**Fig. 2:**
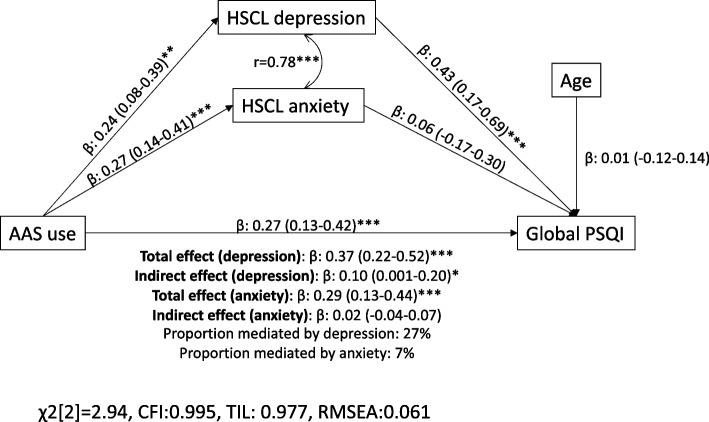
Mediation analysis of Anabolic Androgenic steroid group (AAS), global Pittsburgh Sleep Quality Index (PSQI) and Hopkins symptom checklist (HSCL) for depression and anxiety subscales in addition to age

Spearman correlations showed a robust relationship between the overall PSQI score and the HSCL total (rho = 0.51, *p* < .001), across both groups. Significant correlations were seen for all HSCL and PSQI subscales, as depicted in Fig. S2, with the strongest associations identified between HSCL scales and daytime dysfunction. Both the anxiety and depression subscales exhibited positive associations with the global PSQI scores in both the AAS and in the WLC groups. Detailed results of the correlations analyses for the AAS and WLC group can be found in Fig. S2. Within the AAS group, HSCL, JSS, and PSQI sum scores were not significantly correlated with years of use, dose, or weekly time spent training. Age at initiation of AAS use was moderately negatively correlated with HSCL score (ρ = 0.37, *p* = 0.002). Results from the multiple mediation model is visualized in Fig. 2. There was a significant direct effect of AAS use on sleep. Additionally, an indirect effect for depression was identified, but not for anxiety, suggesting that depression is a partial mediator of the association between AAS use and global PSQI. It should be noted the indirect effect of depression was no longer significant in sensitivity analysis, although the effect was trending (*P* = .07). Results from the sensitivity analysis are presented in Fig. S2.

### Sleep in different phases of anabolic androgenic steroid use and withdrawal

**Fig. 3:**
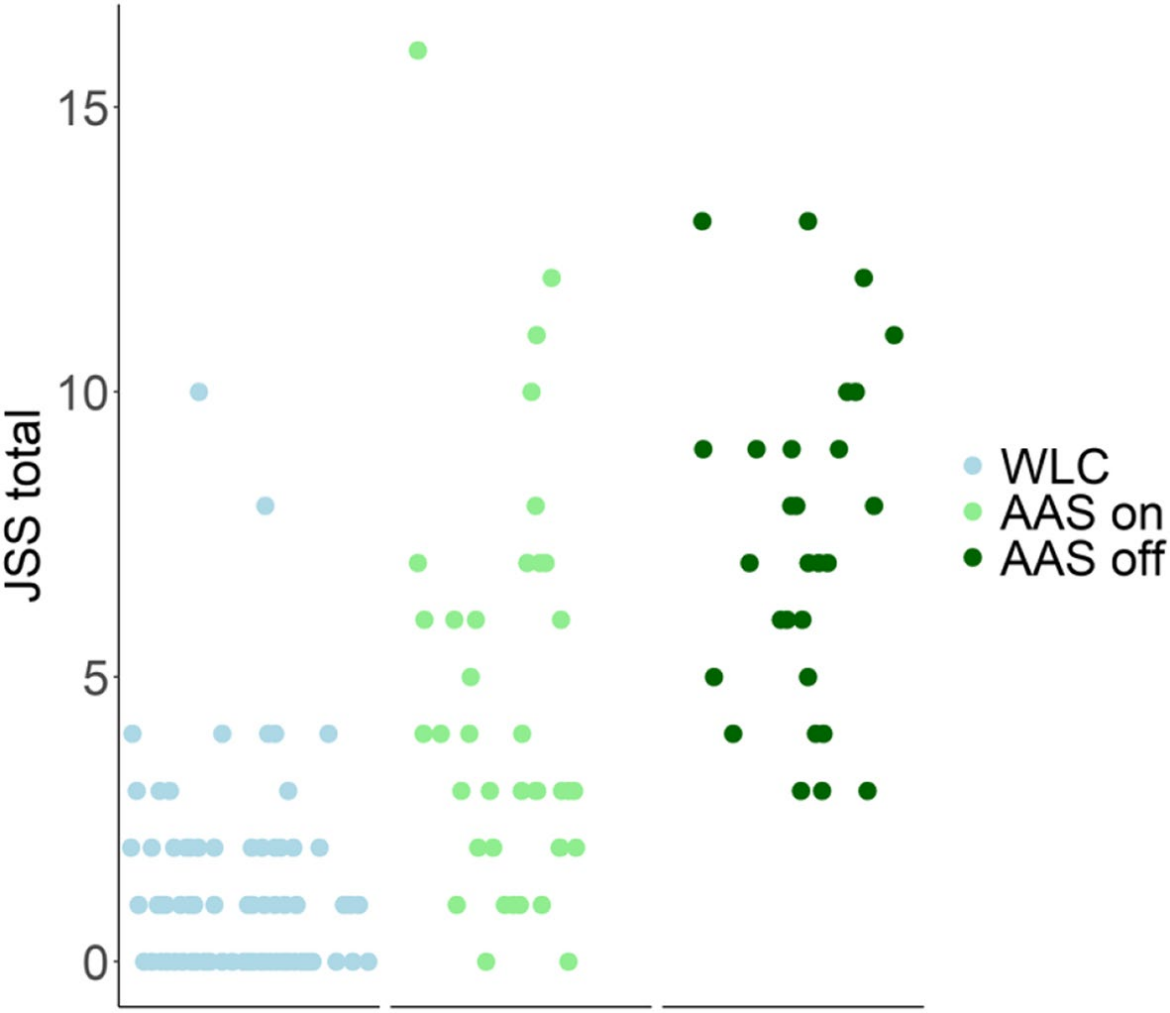
Y axis represents the total JSS score and the x axis presents dates of measurement split in three subplots representing the groups (WLC; blue, AAS on;green and AAS off;dark green). WLC: weightlifting controls, AAS: anabolic androgenic steroid group

See Table 3 for the results from the linear mixed effect model. There was a significant effect for groups, with higher total JSS score for “AAS on” (*p* < .05) and “AAS off” (*p* < .001) when WLC was reference group. Furthermore, a significant mean change in total JSS score was found between “AAS on” and “AAS off” cycle (*p* < .001). See Fig. 3 for a visual representation of JSS scores at different phases of AAS use and in the WLC group.

**Table 3:** Results from linear mixed effect model. WLC: weightlifting controls, AAS: anabolic androgenic steroid group

	*Dependent variable:*
	JSS total
AAS on – AAS off	1.949***
	(0.893, 3.004)
WLC - AAS on	3.573**
	(1.119, 6.027)
WLC - AAS off	5.522***
	(3.045, 7.998)
Constant	1.529*
	(0.095, 2.964)
Observations	157
Log Likelihood	− 332.281
Akaike Inf. Crit.	674.561
Bayesian Inf. Crit.	689.842

^*^p < 0.05; ^**^p < 0.01; ^***^p < 0.001

### Cyclic anabolic androgenic steroid use, sleep and psychological distress

See Table 4 for the results from the linear mixed effect model with the HSCL total score added to the model. There was a significant effect was found for HSCL (*p* < .05). However, the significant effect for group was maintained when adding HSCL to the model, showing higher total JSS scores for the “AAS on” group (*p* < .05) and “AAS off” (*p* < .001) when WLC-group is reference. A significant mean change in total JSS score was found between “AAS on” and “AAS off” (*p* < .001).

**Table 4:** Linear mixed effect model with HSCL total score included. HSCL: Hopkins Symptoms Checklist, WLC: weightlifting controls, AAS: anabolic androgenic steroid group

	*Dependent variable:*
	JSS total
AAS on – AAS off	1.991***
	(0.947, 3.035)
WLC - AAS on	2.697**
	(0.430, 4.063)
WLC - AAS off	4.688***
	(2.399, 6.977)
hscl_sum	3.281**
	(1.237, 5.344)
Constant	−1.995
	(−4.553, 0.562)
Observations	157
Log Likelihood	− 326.892
Akaike Inf. Crit.	665.785
Bayesian Inf. Crit.	684.122

*p < 0.05; **p < 0.01; ***p < 0.001

## Discussion

Although sleep disturbance is a commonly reported side-effect of AAS use, only a few studies have investigated the effects of AAS use on sleep. In a large sample of men who have current or previous long-term AAS use, a significant portion reported sleeping problems and the use of sleep medications. Moreover, in a subsample of men who were monitored during different phases of AAS-administration, we found variations in sleep quality linked with abstinent and on-AAS periods, suggesting that sleep quality may be influenced by hormonal fluctuations. While our analyses revealed strong associations between poor sleep and psychological distress, we found that psychological distress only partially mediated the relations between AAS and sleep. These findings and their potential clinical significance are discussed in further detail below.

### Anabolic androgenic steroid use is associated with poor sleep quality

Our findings are in line with epidemiological studies that have found sleep disturbances to be frequently reported as a side-effect of AAS use [6–9]. Using two widely used assessment tools, we found that the AAS group scored significantly higher than controls on most subscales, besides sleep latency, suggesting that AAS use or associated factors may have a broad impact on different aspects of sleep. The high use of prescribed sleep medications among the AAS group suggests clinically significant sleep disturbances. Additionally, our findings from the cycling subsample revealed that men who use AAS had significantly worse sleep quality compared to the WLC group over time, regardless of administration phase. AAS easily pass the blood–brain barrier and have diverse central nervous system effects, including affecting the mental state [27]. The current findings align with animal and human studies showing that AAS administration can lead to increased energy, agitation, aggressiveness, irritability, affective and psychotic symptoms, which could all impact sleep [28, 29]. Additionally, a placebo-controlled high-dose AAS (250–500 mg/week) trial on healthy older men found shortened sleep duration, lower sleep efficiency, less slow-wave sleep and worsened sleep apnea [16]. Taken together, our findings contribute to increasing evidence showing that AAS use has a negative impact on sleep, and argues for a focus on sleep problems when developing treatment strategies for individuals struggling with AAS misuse.

### Sleep quality is poorer during withdrawal from anabolic androgenic steroids

Research on sleep quality during different phases of AAS administration is limited, but our findings suggest a significant link between sleep disturbances and hormone levels that deviate from physiologically normal levels in both directions. The poorest sleep quality was reported during AAS withdrawal phases, potentially due to a relationship between sleep and anabolic steroid-induced hypogonadism (ASIH) [15, 30]. Amelioration of ASIH symptoms during the administration phase could account for the comparatively improved sleep quality while on cycle. A study by Shigehara et al. (2018) [30] partially supports this notion, demonstrating that testosterone replacement therapy (TRT) can improve sleep conditions in men with hypogonadism. However, direct comparisons between these studies are challenging due to differing study populations and doses. The relationship between sex hormones and sleep is likely complex, involving interactions with other hormones such as cortisol and inflammatory markers [31]. A recent study in a sample partially overlapping with the one presented in this study identified several deviations in hormone levels between men who were on or off AAS cycles. Specifically, the on-cycle group exhibited a distinct hormonal profile compared to both WLC and off-cycle participants, including lower levels of LH, FSH, and SHBG, and elevated serum testosterone level and E2 levels, and where off-cycle or men who had previously used have lower testosterone, SHBG, FSH, LH, and FTI relative to WLC [32]. Longitudinal studies are necessary to further explore the potential role of these mechanisms, specifically monitoring effects of AAS during withdrawal and use. Future studies should investigate the impact of poor sleep quality during withdrawal on AAS dependence, and examine potential increases in the risk of relapse or if interventions to improve sleep quality can prevent relapse, as suggested for substance use disorders [33].

### Links between mental health, anabolic androgenic steroids and sleep

In the current sample, the AAS group reported higher levels of anxiety and depression, which is consistent with earlier studies [34]. In addition, the global PSQI was correlated with all HSCL measures in both the AAS and WLC groups, indicating that the well-established link between sleep and mental health is also present in this sample [17]. Our study found significant links between psychological distress, poor sleep, and AAS use. A direct relationship between AAS use and sleep quality was identified, with psychological distress partially mediating this relationship. This emphasizes the need of targeting sleep problems in treatment strategies of mental health problems, which has been proven to be effective [35]. Furthermore, our results suggest that depression symptoms may play a larger role in the association between AAS use and sleep problems than anxiety. Most likely the relation between sleep and depression is bidirectional. However, previous studies have shown that insomnia proceeded the onset of depression in 69% of cases in comorbid cases of insomnia, anxiety and depression [18]. If sleep problems tend to proceed symptoms of depression in men who use AAS, early intervention and treatment targeting sleep quality could be effective preventive measure for depressive symptoms. The impact of AAS on sleep likely involves complex neurobiological mechanisms. Our previous research has demonstrated decreased functional brain connectivity between the default mode network (DMN) and the amygdala measured at rest among men using AAS [36]. Interestingly, DMN connectivity has been linked with sleep pathologies [37–39]. While directionality cannot be established, disturbed sleep may explain the previously observed decreased functional connectivity among men using AAS. Regardless of the precise nature of the connection, it is evident that sleep disturbances induced by AAS use can have adverse consequences for both physical and mental health.

### Limitations

There current study has several limitations. First, causation cannot be established from the cross-sectional data, limiting the conclusions that can be drawn and low power in the longitudinal cycling-sample limits the generalizability of the longitudinal models. Although the WLC is matched to the AAS group on several aspects, we cannot rule out that AAS use is associated with lifestyle factors that might impact on sleep, such as medical conditions, diet or more alcohol and drug use. Confounding factors such as obesity and sleep apnea known to affect sleep-testosterone relationships were not controlled for in our models [40], and our findings likely reflect something broader than solely the pharmacologic effects of AAS. Indeed, diverse reasons for sleep disturbances were reported by participants in our study, including lifestyle factors, psychological issues, and other causes. Additionally, the self-report nature of our measures for sleep and mental health do not provide detailed information about how different sleep phases are affected by AAS use. Furthermore, the two sleep instruments show huge discrepancy regarding the number of participants above the clinical cut-off. Therefore, although the PSQI and JSS have been deemed adequate screening tools for sleep disturbances in clinical and non-clinical populations [41, 42], there are differences in the level of details provided by the measurements and their definition of clinical threshold for sleep disorders. Also, for the cycling sample, compliance among the participants varied, where the participants’ perseverance might bias the findings towards a certain direction. Lastly, the technical issue where we lack weighted frequency response data from one item, might have biased the PSQI score towards lower scores. While we do not believe that this impacted our group comparisons, it could weaken the generalizability of the PSQI scores. It also important to note that the current study sample of all Norwegian male participants cannot be generalized to other contexts, and the findings should be interpreted with this consideration. Further studies are needed to confirm the observed associations, and preferably studies that includes longitudinal follow ups with a broad spectrum of measurements, including biological markers.

## Conclusion

In conclusion, our findings suggest that AAS use greatly affects sleep, during both use and withdrawal phases. The findings are worrying as sleep pathology can impair mental health and increase vulnerability to illness. Sleep problems may contribute to the increased levels of psychiatric and somatic pathology that sometimes accompany AAS use. Healthcare professionals should thus be made aware that use can impair sleep, so that they can help those who struggle with use and also evaluate whether sleep problems may be a contributing cause of other problems for which they seek help.

### Supplementary Information

**Additional file 1: Table S1. **Sample characteristics and characteristics of AAS use in cycling sample. **Table S2.** Total Jenkins Sleep Scale (JSS) scores and subscales. **Figure S1.** Spearman correlations between each subscale and global scores of PSQI and HSCL scores for all participants (left), AAS (middle) and WLC (right) group. PSQI: Pittsburg Sleep Quality Index, HSCL: Hopkins symptom checklist, WLC: weightlifting controls, AAS: anabolic androgenic steroid group. **Figure S2.** Sensitivity analysis of Anabolic Androgenic Steroid (AAS) use, global Pittsburgh-Sleep-Quality-Index (PSQI) and Hopkins symptoms checklist (HSCL) anxiety and depression score (excluding HSCL_16). **Table S3.** Sensitivity analysis excluding question about sleep in HSCL-10. **Figure S3.** Mediation analysis of Anabolic Androgenic Steroid (AAS) use, global Pittsburgh-Sleep-Quality-Index (PSQI) and Hopkins Symptoms Checklist (HSCL) total score.

## Data Availability

The datasets generated and/or analyzed during the current study are not publicly available due to the sensitive nature of the data but are available from the corresponding author on reasonable request.
