# Supplementary material for: Sleep pathology and use of anabolic androgen steroids among male weightlifters in Norway
Source: BMC Psychiatry. 2024 Jan 22;24:62. doi: 10.1186/s12888-024-05516-6 (PMC10804719; doi:10.1186/s12888-024-05516-6)
Supplement: Supplementary file 1 — Additional file 1: Table S1. Sample characteristics and characteristics of AAS use in cycling sample. Table S2. Total Jenkins Sleep Scale (JSS) scores and subscales. Figure S1. Spearman correlations between each subscale and global scores of PSQI and HSCL scores for all participants (left), AAS (middle) and WLC (right) group. PSQI: Pittsburg Sleep Quality Index, HSCL: Hopkins symptom checklist, WLC: weightlifting controls, AAS: anabolic androgenic steroid group. Figure S2. Sensitivity analysis of Anabolic Androgenic Steroid (AAS) use, global Pittsburgh-Sleep-Quality-Index (PSQI) and Hopkins symptoms checklist (HSCL) anxiety and depression score (excluding HSCL_16). Table S3. Sensitivity analysis excluding question about sleep in HSCL-10. Figure S3. Mediation analysis of Anabolic Androgenic Steroid (AAS) use, global Pittsburgh-Sleep-Quality-Index (PSQI) and Hopkins Symptoms Checklist (HSCL) total score. [file 12888_2024_5516_MOESM1_ESM.pdf]

Appendix

Variable	AAS, N = 11[1]	WLC, N = 11[1]	Test Statistic	p-value[2]
**Age**	34 (14)	37 (10)	0.01	>0.9
**Edu**	15.50 (2.07)	17.18 (2.52)	2.4	0.12
Missing	1	0		
**IQ**	112 (7)	114 (10)	0.01	>0.9
Missing	1	1		
**Height**	184 (8)	178 (7)	3.3	0.070
**Weight**	104 (15)	87 (9)	9.6	0.002
**Strength training (min/week)**	306 (154)	423 (204)	3.5	0.062
Missing	1	0		
**Endurance training(min/week)**	71 (111)	109 (139)	1.5	0.2
Missing	1	0		
**Bench max**	174 (25)	135 (20)	8.9	0.003
Missing	1	3		
**Total years AAS use**	13 (10)	NA (NA)		
**Debut age**	23.6 (7.2)	NA (NA)		
**Anxiety medication** [3]	1 / 10 (10%)	0 / 11 (0%)		
Missing	1	0		
**Antidepressants**[3]	2 / 10 (20%)	0 / 11 (0%)		0.2
Missing	1	0		
**Sleeping medication**[3]	6 / 10 (60%)	0 / 11 (0%)		0.004
Missing	1	0		
**Sleeping problems as side-effect of AAS use**				
No problems	2 / 10 (20%)	0 / 0 (NA%)		
Some degree	6 / 10 (60%)	0 / 0 (NA%)		
Certain degree	2 / 10 (20%)	0 / 0 (NA%)		

AAS: Anobolic Androgenic stereoids
[1]Mean (SD); n / N (%)

[2]Kruskal-Wallis rank sum test; Fisher's exact test
[3]Prescribed medication

Table S1: Sample characteristics and characteristics of AAS use in cycling sample

Variable	**WLC**, N = 58[1]	**AAS**, N = 68[1]	Test Statistic	p-value[2]	Kruskal ES
**JSS total**	3.8 (3.6)	7.2 (4.9)	17	<0.001	0.129
**JSS 1**	0.81 (1.16)	1.35 (1.57)	3.8	0.051	0.023
**JSS 2**	1.31 (1.38)	2.29 (1.89)	8.9	0.003	0.064
**JSS 3**	0.76 (1.01)	1.69 (1.66)	12	<0.001	0.086
**JSS 4**	0.95 (1.07)	1.84 (1.56)	11	0.001	0.079
**JSS total>12**	3 (5.2%)	11 (16%)		0.085	

JSS: Jenkins Sleep Scale

[1]Mean (SD); n (%)

[2]Kruskal-Wallis rank sum test; Fisher's exact test

Table S2: Total Jenkins Sleep Scale (JSS) scores and subscales

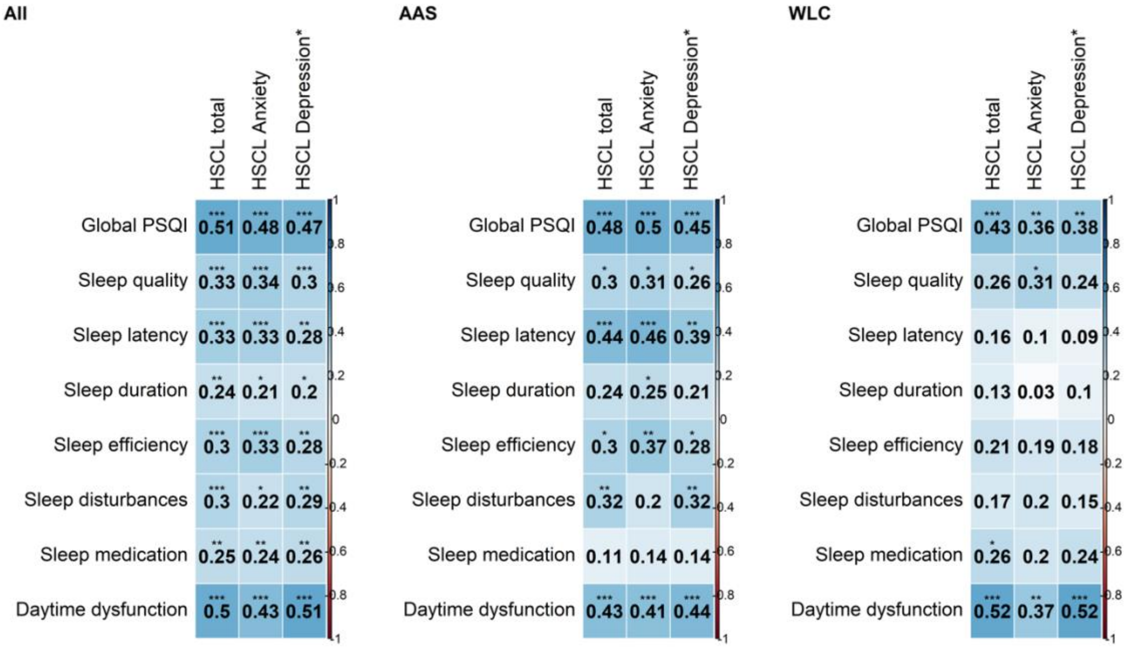

Figure S1: Spearman correlations between each subscale and global scores of PSQI and HSCL scores for all participants (left), AAS (middle) and WLC (right) group. PSQI: Pittsburg Sleep Quality Index, HSCL: Hopkins symptom checklist, WLC: weightlifting controls, AAS: anabolic androgenic steroid group.

*HSCL_16 removed

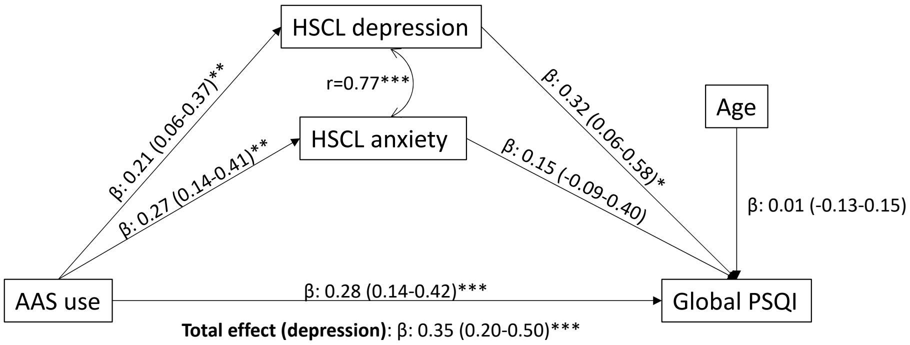

Total effect (depression): β: 0.35 (0.20-0.50)***
Indirect effect (depression): β: 0.07 (-0.02-0.15)
Total effect (anxiety): β: 0.31 (0.17-0.46)***
Indirect effect (anxiety): β: 0.03 (-0.02-0.09)
Proportion mediated by depression: 20.0%
Proportion mediated by anxiety: 9.7%

χ2[2]=3.29, CFI:0.993, TIL: 0.967, RMSEA:0.071

Figure S2:Sensitivity analysis of Anabolic Androgenic Steroid (AAS) use, global Pittsburgh-Sleep-Quality-Index (PSQI) and Hopkins symptoms checklist (HSCL) anxiety and depression score (excluding HSCL_16)

	Dependent variable:
	sleep_sum
AAS on – AAS off	2.048***
	(0.985, 3.111)
WLC- AAS on	3.055**
	(0.729, 5.381)
WLC- AAS off	5.103***
	(2.774, 7.433)
hscl_sum without #16	2.058
	(-0.003, 4.119)
Constant	-0.669
	(-3.204, 1.867)
Observations	157
Log Likelihood	-329.553
Akaike Inf. Crit.	671.106
Bayesian Inf. Crit.	689.444
Note:	*p<0.05; **p<0.01; ***p<0.001

Table S3: Sensitivity analysis excluding question about sleep in HSCL-10

We ran a Lavaan mediation model, the total effect was 0.39 (CI 95%: 0.25, 0.53, p <.001). The mediation effect was 0.13 (CI95%: 0.04, 0.21, p =0.004), accounting for 33% of the total effect. Standardized estimates in the path model for the path from Group to global PSQI were 0.27 (95%CI: 0.12, 0.41, p<.001), accounting for 67% of the total effect.

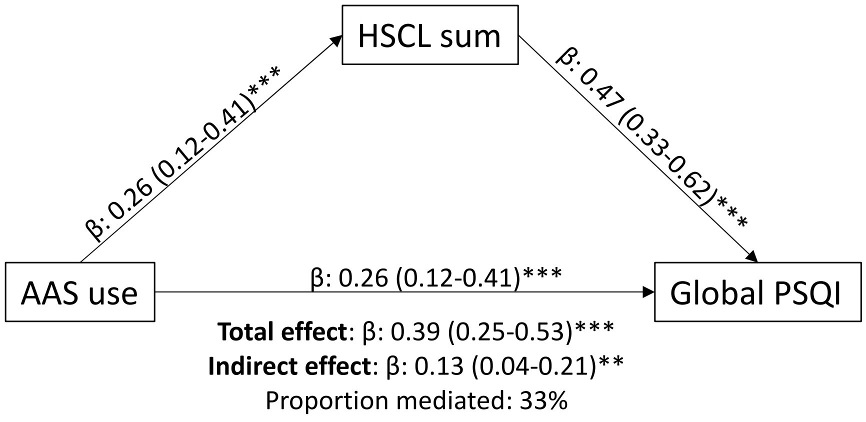

Figure S 3: Mediation analysis of Anabolic Androgenic Steroid (AAS) use, global Pittsburgh-Sleep-Quality-Index (PSQI) and Hopkins Symptoms Checklist (HSCL) total score
